# Subjective outcomes 12 years after transvaginal mesh versus native tissue repair in women with recurrent pelvic organ prolapse; a randomized controlled trial

**DOI:** 10.1007/s00192-022-05442-9

**Published:** 2023-01-20

**Authors:** Kirsten B. Kluivers, Metteke Kamping, Alfredo L. Milani, Joanna IntHout, Mariella I. Withagen

**Affiliations:** 1 Department of Obstetrics & Gynecology (623), Radboud University Medical Center, P.O. Box 9101, 6500 HB Nijmegen, the Netherlands; 2 Department of Obstetrics & Gynaecology, Reinier de Graaf Gasthuis, P.O. Box 5011, 2600 GA Delft, the Netherlands; 3 Department for Health Evidence, Radboud Institute for Health Sciences, Radboud University Medical Center, P.O. Box 9101, 6500 HB Nijmegen, the Netherlands; 4 Department of Obstetrics & Gynecology, University Medical Center Utrecht, Heidelberglaan 100, Room F05.126, P.O. Box 85500, Utrecht, the Netherlands

**Keywords:** Pelvic organ prolapse, Non-absorbable vaginal mesh, Native tissue repair, Subjective outcome

## Abstract

**Introduction and hypothesis:**

The present study describes an extended follow-up study after 12 years and focusses on subjective outcomes of women who underwent surgery for recurrent pelvic organ prolapse in the randomized index study.

**Methods:**

One hundred and ninety-four (194) women had been randomized in the original study and in the present study, 45 (47%) in the vaginal mesh repair versus 43 (43%) women with conventional vaginal native tissue repair completed the long-term questionnaires. The mesh used was a first-generation non-absorbable mesh kit. All types of conventional vaginal native tissue repairs were allowed, and additional vaginal native tissue repairs were allowed in the mesh group. The questionnaires as applied at baseline were used. The Patient Global Impression of Improvement questionnaire (PGI-I) was the primary outcome.

**Results:**

At 12 years, 30 (71%) women in the mesh group versus 23 (59%) women in the native tissue repair group reported to be PGI-I (very) much improved (*p*=0.24). There were no differences found in any of the questionnaire domains. There was, however, a higher number of women who had had additional operations for recurrent pelvic organ prolapse, stress urinary incontinence, and/or exposure in the mesh group: 18 women (40%) in the mesh group versus 8 women (19%) in the native tissue repair group (*p*=0.03).

**Conclusions:**

There was no difference in subjective outcome between the groups, but there was a statistically significant higher number of women who had needed further operations. This study confirms that vaginal mesh should not be used in all women with recurrent pelvic organ prolapse.

## Introduction

Pelvic organ prolapse (POP) is a common health problem worldwide and has a high impact on quality of life [1]. Treatment outcomes of reconstructive surgery are suboptimal. The 10-year rate of repeat surgery for POP and urinary incontinence (UI) is between 1 out of 6 to 10 women [2, 3]. Synthetic mesh was introduced in vaginal POP surgery in France in 2002 with the aim of more durable and effective treatment results [4]. The worldwide implementation of mesh kits preceded robust clinical trials and resulted in high numbers of unexpected adverse events [5, 6]. Many vaginal mesh products have now been removed from the market and regulations vary per country.

Mesh exposure and severe pain/dyspareunia are much feared long-term complications of vaginal mesh surgery [7]. Mesh exposure is a complication exclusively after mesh surgery, but pain complaints, and especially dyspareunia, may also be seen after native tissue repairs for POP. This evidence is, however, less clear. One out of six women after native tissue repair was prescribed opioids for more than 3 months within the first half year after POP surgery [8]. On the other hand, the OPTIMAL trial has shown us that pain scores decreased after native tissue POP surgery up until at least 2 years after surgery [9].

A limited number of randomized controlled trials (RCT) compared the safety and efficacy of synthetic non-absorbable mesh with native tissue repair in daily clinical practice (i.e., mixed patient groups, with both primary and recurrent POP) [10]. Long-term data are scarce, although the PROSPECT study now provides 6 years of follow-up data [3]. The present study focuses on subjective outcomes of the extended follow-up study after 12 years of women who underwent surgery for recurrent POP and were randomized to either vaginal mesh or conventional native tissue repair.

## Materials and methods

The original trial [10] was performed in 13 centers in The Netherlands between August 2006 and July 2008 (www.clinicaltrials.gov, NCT00372190). Patients with recurrent POP were randomized 1:1 between vaginal mesh repair and conventional vaginal native tissue repair. The mesh used in this trial was the first-generation trocar-guided non-absorbable tension-free vaginal mesh (TVM) (Prolift™, Ethicon, Somerville, NJ, USA). This is a macroporous, monofilament, polypropylene mesh with a mesh of 45 g/m^2^. The anterior, posterior, or total mesh version was used. Additional conventional vaginal native tissue repairs were allowed and comprised anterior or posterior colporrhaphies; Manchester procedure; or vaginal hysterectomy with high uterosacral ligament suspension, sacrospinous ligament suspension, or a combination of the latter three procedures. Details regarding design, randomization, sample size, surgical interventions, and 1- and 7-year outcomes have previously been published [10, 11].

An extended follow-up was approved by the Medical Ethics Committee, region Arnhem-Nijmegen, The Netherlands, on 28 April 2014 under no. NL46834. 091.14 and (www.clinicaltrials.gov, NCT00372190). Informed consent was obtained prior to inclusion in the follow-up study. The protocol included physical examination and questionnaires at 7 years’ follow-up [11] and a questionnaire at 10 years’ follow-up. The present follow-up study was planned and performed as a questionnaire study only. Measurements were delayed owing to low work force capacity and resulted in 12 years’ follow-up. This postponement was approved as an amendment of the study protocol in January 2020 by the same ethics committee.

All participants completed the Dutch version of the validated questionnaires: Patient Global Impression of Improvement questionnaire (PGI-I), EuroQol-5D (EQ-5D), Urogenital Distress Inventory (UDI), Defecatory Distress Inventory (DDI), Incontinence Impact Questionnaire (IIQ), and Visual Analogue Scale (VAS) pain score [12–15]. The questionnaires, besides PGI-I and VAS, had also been completed at baseline, 12 years previously. At baseline, the PISQ-12 score [16] had furthermore been assessed, but this questionnaire was not distributed. Reasons were the low numbers of sexually active women, the low response rate to intimate questions at the previous follow-up, and the fact that there had not been any change over time since baseline.

The PGI-I is a seven-point Likert scale to rate the patient-reported response of a condition (prolapse) to therapy (surgery), where 1 = very much better, 2 = much better, 3 = a little better, 4 = no change, 5 = a little worse, 6 = much worse, and 7 = very much worse. These mean scores are presented per treatment group as well as the percentage of women reporting their improvement very much better or much better. The EQ-5D records the patient’s self-rated health on a five-point scale (excellent, very good, good, fair, or poor). The UDI, DDI, and IIQ are each subdivided into five domains, with subscales ranging from 0 to 100 and higher scores indicating more bother and worse QoL. The VAS pain ranged from 0 to 10 and was assessed as spontaneous pain and pain on activity.

The primary outcome of this extended follow-up study was defined as the difference in PGI-I score between the mesh group and the native tissue repair group. Secondary outcomes were patient-reported retreatment for POP, SUI, and mesh exposure, and further questionnaire scores.

Missing data of women who were lost at 12-year follow-up were not imputed. Analysis was carried out according to the intention-to-treat principle. Per protocol analysis was performed to rule out a dilution of real effects due to mesh surgery during repeat surgery in the native tissue repair group. Descriptive statistics with mean scores and standard deviations and numbers with percentage were used to summarize outcomes. Statistical significance was analyzed using independent samples *t* tests or Chi-squared tests as appropriate. Statistical analyses were performed using SPSS Statistics for Windows, version 26.0.

## Results

**Fig. 1:**
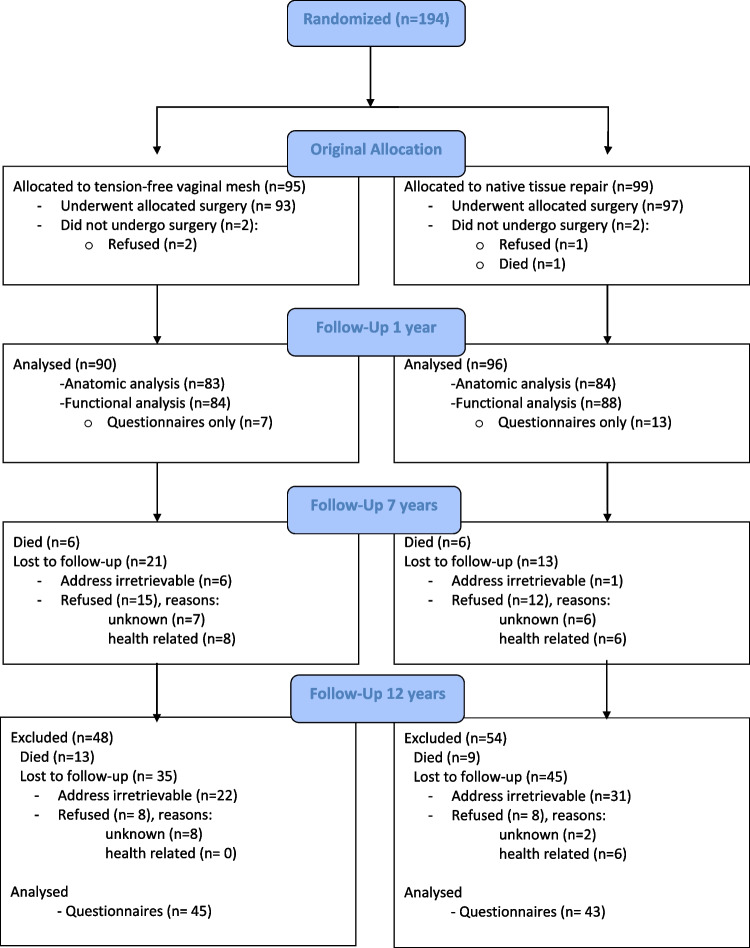
Flowchart of randomization and follow-up

One hundred and ninety-four women were randomized in the index trial, of whom 95 (49%) to surgery using mesh and 99 (51%) to native tissue repair. In the present study, 45 women (47%) versus 43 women (43%) completed the long-term questionnaires (see flowchart in Fig. 1).

Baseline characteristics (age, parity, BMI, and overall POP stage) of participants in the 12-year follow-up are similar in the mesh and native tissue repair groups. The index operations by treatment group are reported in Table 1, which shows that almost half of the patients had a posterior mesh in the mesh group. The responders to the questionnaires in this 12-year follow up had been younger at baseline than the non-responders (61.1 ± 8.6 versus 65.9 ± 11.1 years of age during the index surgery; *p*=0.01). At the 7-year follow-up [11] the responders had lower POP stages at baseline (at the time of the index surgery), but this difference was not seen at 12 years.

**Table 1:** Index operations in the mesh and native tissue repair groups

Index operation	Mesh group (*n*=45)	Native tissue group (*n*=43)
Anterior mesh	18 (40%)	n/a
Posterior mesh	22 (49%)	n/a
Total mesh	5 (11%)	n/a
Sacrospinous fixation	2^a^	16 (37%)^b^
Modified Manchester	1^a^	2 (5%)^b^
Vaginal hysterectomy		2 (5%)^b^
Anterior repair	2^a^	9 (21%)
Posterior repair		12 (28%)
Anterior/posterior repair		2 (5%)
Vaginal enterocele repair		1 (2%)

*n/a* not applicable

^a^In the mesh group an anterior mesh was combined with a sacrospinous fixation in 2 cases and a Modified Manchester in 1 case, a posterior mesh was combined with a native tissue anterior repair in 2 cases and one total mesh was combined with a midurethral sling

^b^In the native tissue repair group sacrospinous fixation was combined with anterior repair (*n*=4), posterior repair (*n*=3), anterior/posterior repair (*n*=8) or vaginal enterocele repair (*n*=1). Modified Manchester was combined with anterior repair (*n*=2). Vaginal hysterectomy was combined with anterior repair (*n*=1) and posterior repair (*n*=1)

The PGI-I was completed by 42 women (93%) in the mesh group and 39 women (91%) in the native tissue repair group. Table 2 shows the mean questionnaire scores and the differences over the period of 12 years. There is no statistically significant difference in PGI-I score between the groups (primary outcome: 2.5 ± 1.5 versus 2.6 ± 1.2 in mesh versus native tissue repair group, *p*=0.19), nor was there a statistically significant difference between groups in the number of women who reported to be (very) much improved after surgery. No statistically significant differences in the subjective outcomes between the groups were detected and this did not change when applying a per protocol analysis.

**Table 2:** Questionnaire scores at 12 years and the change over time in the mesh and native tissue repair group

Questionnaire	Mesh group (*n*=45)		Native tissue group (*n*=43)		*p*
	Score 12 years	Change	Score 12 years	Change	
PGI-I	2.5 ± 1.5	n/a	2.6 ± 1.2	n/a	.19
PGI-I (very) much improved	30/42 (71%)	n/a	23/39 (59%)	n/a	.24
VAS spontaneous pain	1.0 ± 1.2	n/a	1.5 ± 2.4	n/a	.17
VAS pain on activity	1.7 ± 1.3	n/a	1.5 ± 3.0	n/a	.48
EQ-5D	4.5 ± 1.2	0 ± 1.4	4.4 ± 1.2	−0.1 ± 1.5	.59
UDI					
Overactive bladder	28.2 ± 26.9	−3.8 ± 30.5	30.0 ± 27.7	0.6 ± 26.1	.22
Incontinence	25.8 ±26.1	−1.2 ± 22.8	24.0 ± 24.5	0.4 ± 24.6	.86
Obstructive micturition	14.8 ± 24.4	−7.4 ± 28.9	23.9 ± 27.7	−1.3 ± 28.8	.48
Pain	14.3 ± 24.3	−9.3 ± 29.8	18.8 ± 25.7	−10.1 ± 31.2	.89
Genital prolapse	8.3 ± 17.0	−39.2 ± 36.5	7.0 ± 20.0	−40.6 ± 28.8	.19
DDI					
Constipation	13.6 ± 22.8	0.9 ± 23.6	8.5 ± 16.0	0.5 ± 19.3	.51
Obstructive defecation	14.9 ± 21.6	1.0 ± 20.3	10.5 ± 15.8	−4.3 ± 17.7	.65
Pain	4.5 ± 12.6	−5.0 ± 18.0	8.5 ± 20.7	−2.1 ± 18.4	.88
Fecal incontinence	13.6 ± 23.4	6.1 ± 20.7	13.6 ± 18.6	0.8 ± 25.7	.79
Flatus	45.2 ± 33.6	0 ± 35.3	43.9 ± 36.5	4.8 ± 32.6	.77
IIQ					
Physical	11.2 ± 21.1	−6.1 ± 31.8	6.6 ± 15.1	−14.5 ± 25.2	.63
Mobility	17.8 ± 22.1	−4.8 ± 30.4	20.6 ± 25.8	−5.4 ± 24.0	.46
Social	7.5 ± 12.9	−8.8 ± 18.1	6.1 ± 10.1	−11.9 ± 17.7	.81
Embarrassment	13.3 ± 19.3	−0.5 ± 19.0	13.3 ± 21.3	−3.3 ± 26.8	.33
Emotional	12.7 ± 15.3	−7.4 ± 21.2	15.0 ± 20.8	−5.6 ± 20.1	.92

Data are presented as mean score at 12 years and mean changes (between baseline and 12-year scores) ± standard deviation (SD) or numbers (percentage of responders) per intervention group

*Change* score change between 12 years minus baseline measurement, *p* *p* value, where available this was the *p* value for the mean difference in change since baseline, and for the absolute score at 12 years otherwise, between the mesh and native tissue repair groups, as calculated by independent samples *t* test, or Chi-squared test for the numbers (percentage), *PGI-I* Patient Global Impression of Improvement (ranges from 1 to 7, where 1 denotes most improvement) (PGI-I (very) much improved = women reporting to be very much improved or much improved), *VAS* visual analog scale score (ranges from 0 to 10, where 10 denotes most pain), *EQ-5D* EuroQol group questionnaire (ranges from 1 to 6, where 1 denotes the poorest health status), *UDI* Urogenital Distress Inventory, *DDI* Defecatory Distress Inventory, *IIQ* Incontinence Impact Questionnaire (domain scores UDI, DDI, and IIQ range from 0 to 100, with lower scores representing better outcome. A negative score for change over time reflects a reduction in bother and improved quality of life compared with baseline), *n/a* not applicable

Fourteen women (31%) in the mesh group reported in the questionnaire that they had had a treated or nontreated exposure since the index surgery (versus none in the native tissue repair group).

One further mesh excision for exposure was reported in the mesh group for the period between 7 and 12 years’ follow-up. Among the participants in this 12-year follow-up, there were 5 mesh excisions in the mesh group (11%) and 1 suture exposure after sacrospinous hysteropexy in the native tissue repair group (2%) cumulative since the index surgery (*p*=0.11). Index operations in women with exposure were an anterior mesh in 1 case, total vaginal mesh in 2 cases, and posterior mesh in 2 cases. Five women in the native tissue repair group at 12 years’ follow-up have received at least one surgical implant (i.e., 2 anterior meshes, 1 posterior mesh, 3 midurethral slings, and 1 sacral colpopexy) since the index surgery. None of these women needed an operation for exposure.

Three women reported POP surgery for recurrence of POP during the period between 7 and 12 years’ follow-up; 1 (2%) in the mesh group and 2 (5%) in the native tissue repair group: a posterior colporrhaphy in an untreated compartment in the mesh group, and an anterior mesh placement (treated compartment) and sacral colpopexy (untreated compartments) in the native tissue repair group. Since the index surgery, 12 women (27%) in the mesh group, versus 6 (14%) in the native tissue repair group had repeat surgery for POP (*p*=0.14). Since the index surgery, 5 women (11%) in the mesh group received a midurethral sling, of whom 3 had POP surgery as well, versus 3 women (7%) in the native tissue repair group received a midurethral sling, of whom 2 had repeat surgery for POP as well (*p*=0.10). When adding up the additional operations for recurrent POP, the midurethral slings and the exposure excisions, 18 women (40%) in the mesh group versus 8 women (19%) in the native tissue repair group have had additional surgery (*p*=0.03). Table 3 shows the data above on reoperations for POP, SUI, and excision of exposures by treatment group. Further pelvic surgery was performed in 1 woman (2%) in the mesh group (neurostimulator implantation for overactive bladder), and 3 women (7%) in the native tissue repair group (rubber band ligation, anal abscess/fistula, and rectal carcinoma).

**Table 3:** Number of women who had an operation in the mesh versus native tissue repair group by follow-up period

Number of women who underwent	Mesh group (*n*=45)			Native tissue group (*n*=43)			*p*
	0–7 years	7–12 years	0–12 years	0–7 years	7–12 years	0–12 years	
Repeat operation for POP	11 (24%)	1 (2%)		4 (9%)	2 (5%)		0.14
SUI operation	5 (11%)			2 (5%)	1 (2%)		0.10
Excision exposure	4 (9%)	1 (2%)		1 (2%)	0		0.11
Operation for POP, SUI and/or exposure^a^			18 (40%)			8 (19%)	0.03

Data presented as number of women (and percentage of responders) per intervention group and by time period

*POP* pelvic organ prolapse, *SUI* stress urinary incontinence, *p* *p* value for the difference between the number of women who had the operation in the mesh and native tissue repair groups during the 12 years’ follow-up period, as calculated by independent samples Chi-squared test for the intention to treat

^a^Note that some women had more than one operation

## Discussion

The 12-year follow-up of a multicenter RCT including women with recurrent POP undergoing trocar-guided transvaginal non-absorbable mesh insertion versus native tissue repair showed similar patient-reported global impression of improvement between groups. Two-thirds of women experienced (very) much improvement after treatment.

Forty percent of women randomized to mesh surgery had repeat surgery for either POP, stress urinary incontinence, or exposure during the 12-year follow-up. This is a doubled risk compared with the native tissue repair group. The difference is statistically significant as well as clinically relevant. The difference was already seen as a trend at 7 years’ follow-up. Women treated with mesh in a single vaginal compartment continued to need POP surgery in the nontreated compartment(s), as was seen after 7 years’ follow-up [11, 17, 18]. In contrast, the repeat surgeries in the native tissue repair group were equally divided between in the treated and untreated vaginal compartment.

The improvement in pelvic floor-specific symptoms since baseline was most marked in the UDI domain genital prolapse. This shows a long-lasting positive effect of POP surgery, despite high repeat surgery rates. The improvement in the UDI domain pain and the low VAS pain scores at rest and activity in both groups are remarkable in the light of the pain complications reported after vaginal mesh insertions in the media [6]. These outcomes on pain support previous results from the OPTIMAL study, where a 2-year lasting decrease in pain scores was reported after uterosacral and sacrospinous vaginal vault suspension [9].

No major mesh excisions were needed in this study sample. There were 5 (11%) new patient-reported exposures since the 7-year follow-up, of which 1 patient had a mesh excision for exposure (2%). During the 12 years, 18 out of 45 women in the mesh group (40%) had an exposure since the index surgery (versus 1 (3%) suture exposure after sacrospinous fixation in the native tissue repair group). At 7 years, the sample size of the mesh group was 53 women, of whom 22 (42%) had an exposure, with repeat surgery for mesh exposure in 7 women (13%). The next-generation meshes have already been adapted to decrease the high incidence of exposures seen with the use of this non-absorbable and relatively large and heavy mesh [19]. Whether these meshes have better outcomes and acceptable exposure rates in the long term will need careful assessment.

A clear strength of this study is the long follow-up. The response rate is almost half of the randomized women and this resulted in a small sample. The response rate may still be regarded as acceptable in a trial on POP surgery. Although the response rate in long-term studies on urinary incontinence treatments is generally higher [20], this cannot be expected from studies on women having recurrent POP who are older. The nonresponders in the present trial would have been 78 years of age at the 12-year follow-up, and it may not be feasible to complete a questionnaire at that age. There might be some unknown selection bias in the study when, for example, women with more extreme outcomes do or do not respond. When comparing the 12-year results with the results from the previous follow-up moments, there seem to be no significant trend breaks in the outcomes of the women included in this RCT.

“Composite” outcomes could not be calculated that combine anatomical success with the absence of bulge symptoms in the absence of repeat treatment for POP, and which are considered clinically most relevant [21]. It was for the sake of feasibility of this study in 13 different centers, that we decided to assess the subjective outcomes only. This differs from the previous studies, where anatomical outcomes were collected as well.

The studied mesh kit was the first version of the Gynecare Prolift™ Pelvic Floor Repair system, which was withdrawn from the market in 2013. According to sales information by Johnson & Johnson, this mesh system has potentially been used in over 220,000 women worldwide. It is only after this careful long-term evaluation of efficacy and safety that we would be able to counsel women on the pros and cons of this specific product [22].

In conclusion, the current study demonstrates that POP operations with the use of this vaginal mesh kit and native tissue repairs were able to reach stable long-term outcomes. Two-thirds of women felt (very) much improved and there were no relevant differences in patient-reported outcomes between groups. There was, however, a doubled number of women in the vaginal mesh group who had undergone at least one further operation for recurrent POP, stress urinary incontinence, and/or exposure. The previously observed trend with more recurrent POP surgeries in the nonmesh-augmented vaginal compartments continued in the long term. No clear benefit of mesh was seen in this study. This confirms that vaginal mesh should not be used in all women with recurrent POP.

## References

[1] Wu JM, Hundley AF, Fulton RG, Myers ER (2009). Forecasting the prevalence of pelvic floor disorders in U.S. women: 2010 to 2050. Obstet Gynecol.

[2] Denman MA, Gregory WT, Boyles SH, Smith V, Edwards SR, Clark AL (2008). Reoperation 10 years after surgically managed pelvic organ prolapse and urinary incontinence. Am J Obstet Gynecol.

[3] Reid FM, Aucott L, Glazener CMA, Elders A, Hemming C, Cooper KG (2022). PROSPECT: 4- and 6-year follow-up of a randomised trial of surgery for vaginal prolapse. Int Urogynecol J.

[4] Debodinance P, Berrocal J, Clave H (2004). Changing attitudes on the surgical treatment of urogenital prolapse: birth of the tension-free vaginal mesh. J Gynecol Obstet Biol Reprod (Paris).

[5] UPDATE on Serious Complications Associated with Transvaginal Placement of Surgical Mesh for Pelvic Organ Prolapse: FDA Safety Communication. July 13, 2011. http://www.fda.gov/MedicalDevices/Safety/AlertsandNotices/ucm262435.htm 10.1007/s00192-011-1581-2 22086260

[6] Fadaee N, Huynh D, Towfigh S (2020). #mesh: social media and its influence on perceptions in hernia repair. Am Surg.

[7] Maher C, Feiner B, Baessler K, Christmann-Schmid C, Haya N, Marjoribanks J (2016). Transvaginal mesh or grafts compared with native tissue repair for vaginal prolapse. Cochrane Database Syst Rev.

[8] Gressler LE, dosReis S, Chughtai B (2021). Opioid prescribing and risks among commercially insured women undergoing pelvic organ prolapse repair. Pharmacoepidemiol Drug Saf.

[9] Barber MD, Brubaker L, Nygaard I, Wai CY, Dyer KY, Ellington D (2019). Pain and activity after vaginal reconstructive surgery for pelvic organ prolapse and stress urinary incontinence. Am J Obstet Gynecol.

[10] Withagen MI, Milani AL, den Boon J, Vervest HA, Vierhout ME (2011). Trocar-guided mesh compared with conventional vaginal repair in recurrent prolapse: a randomized controlled trial. Obstet Gynecol.

[11] Milani AL, Damoiseaux A, IntHout J, Kluivers KB, Withagen MIJ (2018). Long-term outcome of vaginal mesh or native tissue in recurrent prolapse: a randomized controlled trial. Int Urogynecol J.

[12] Srikrishna S, Robinson D, Cardozo L (2010). Validation of the patient global impression of improvement (PGI-I) for urogenital prolapse. Int Urogynecol J.

[13] EuroQol Group (1990). EuroQol—a new facility for the measurement of health-related quality of life. Health Policy.

[14] Van der Vaart CH, de Leeuw JR, Roovers JP, Heintz AP (2003). Measuring health-related quality of life in women with urogenital dysfunction: the urogenital distress inventory and incontinence impact questionnaire revisited. Neurourol Urodyn.

[15] Price DD, Patricia A, McGrath PA, Rafii A, Buckingham B (1983). The validation of visual analogue scales as ratio scale measures for chronic and experimental pain. Pain.

[16] ’t Hoen LA, Utomo E, Steensma AB, Blok BF, Korfage IJ (2015). The pelvic organ prolapse/urinary incontinence sexual questionnaire (PISQ-12): validation of the Dutch version. Int Urogynecol J.

[17] Withagen MI, Vierhout ME, Milani AL (2010). Does trocar-guided tension-free vaginal mesh (Prolift) repair provoke prolapse of the unaffected compartments?. Int Urogynecol J.

[18] Withagen MI, Milani AL, de Leeuw JW, Vierhout ME (2012). Development of de novo prolapse in untreated vaginal compartments after prolapse repair with and without mesh: a secondary analysis of a randomised controlled trial. BJOG.

[19] Dykes N, Karmakar D, Hayward L (2020). Lightweight transvaginal mesh is associated with lower mesh exposure rates than heavyweight mesh. Int Urogynecol J.

[20] Bakas P, Papadakis E, Karachalios C, Liapis I, Panagopoulos N, Liapis A (2019). Assessment of the long-term outcome of TVT procedure for stress urinary incontinence in a female population: results at 17 years' follow-up. Int Urogynecol J.

[21] Barber MD, Brubaker L, Nygaard I (2009). Defining success after surgery for pelvic organ prolapse. Obstet Gynecol.

[22] Farquhar C (2016). No implementation without evaluation: the case of mesh in vaginal prolapse surgery. Cochrane Database Syst Rev.

